# Endoscopic imaging of white matter fiber tracts using polarization-sensitive optical coherence tomography

**DOI:** 10.1016/j.neuroimage.2022.119755

**Published:** 2022-11-15

**Authors:** Damon DePaoli, Daniel C. Côté, Brett E. Bouma, Martin Villiger

**Affiliations:** a Harvard Medical School, Boston, MA 02115, USA; b Wellman Center for Photomedicine, Massachusetts General Hospital, Boston, MA 02114, USA; c CERVO Brain Research Center, Université Laval, Quebec City, Quebec G1E 1T2, Canada; d Institute for Medical Engineering and Science, Massachusetts Institute of Technology, Cambridge, MA 02142, USA

**Keywords:** Optical coherence tomography, Polarized light imaging, Optical anisotropy, Birefringence, Intracranial imaging

## Abstract

Polarization sensitive optical coherence tomography (PSOCT) has been shown to image and delineate white matter fibers in a label-free manner by revealing optical birefringence within the myelin sheath using a microscope setup. In this proof-of-concept study, we adapt recent advancements in endoscopic PSOCT to perform depth-resolved imaging of white matter structures deep inside intact porcine brain tissue *ex-vivo*, through a small, rotational fiber probe. The probe geometry is comparable to microelectrodes currently used in neurosurgical interventions. The presented imaging system is mobile, robust, and uses biologically safe levels of optical radiation making it well suited for clinical translation. In neurosurgery, where accuracy is imperative, endoscopic PSOCT through a narrow-gauge fiber probe could provide intra-operative feedback on the location of critical white matter structures.

## 1. Introduction

Despite the inherent risk associated, neurosurgery is a necessary procedure for the treatment of a multitude of neurologic disorders. To both mitigate iatrogenic damage and target deep brain structures, magnetic resonance imaging (MR, MRI) is the traditional method of choice for pre-surgical imaging and planning. More recently, diffusion magnetic resonance imaging (dMRI), which allows the visualization of white matter architecture, has shown promise for improved surgical planning, ameliorating treatment outcomes in a broad range of neurosurgical procedures (Coenen et al., 2018; Chen et al., 2007; Barbosa et al., 2016).

Real-time intraoperative feedback can also be advantageous for a surgeon to both improve on the planned procedure and to correct for deviations from the planned targeting. Intraoperative MRI has shown promise for providing such real-time feedback during neurosurgery but requires a dedicated operating room with compatible equipment. Furthermore, current intraoperative MR systems often cannot provide sufficient resolution for direct visualization of intracranial instruments and their position relative to neuroanatomical features. Such information could facilitate enhanced intra-surgical guidance. Therefore, optical imaging modalities that can provide similar contrast to MR modalities, but with higher resolution and in a cost-effective manner may be promising complementary tools within the clinical workflow.

The recent advances in dMRI have shown that white matter tracts (WMT) can provide useful anatomical landmarks for improving neurosurgical planning (Essayed et al., 2017). The fibrillar architecture of WMT not only leads to the anisotropic diffusion underlying dMRI, but also induces optical anisotropy (de Campos Vidal et al., 1980). This manifests as birefringence, whereby light experiences two slightly different refractive indices when polarized along or orthogonal to the tissue fiber direction, i.e., its optic axis orientation. Polarized light imaging (PLI) (Axer et al., 2011) and polarization-sensitive optical coherence tomography (PSOCT) (Wang et al., 2016) have been exploiting this effect to provide label-free, high-resolution imaging of WMT *ex vivo*. Microscopic PSOCT has demonstrated delineation of WMT in both fixed mouse (Wang et al., 2016; Nakaji et al., 2008) and human brain tissue (Wang et al., 2016; Wang et al., 2014), and has served to decode the microstructural correlate of dMRI (Jones et al., 2020).

**Fig. 1.**
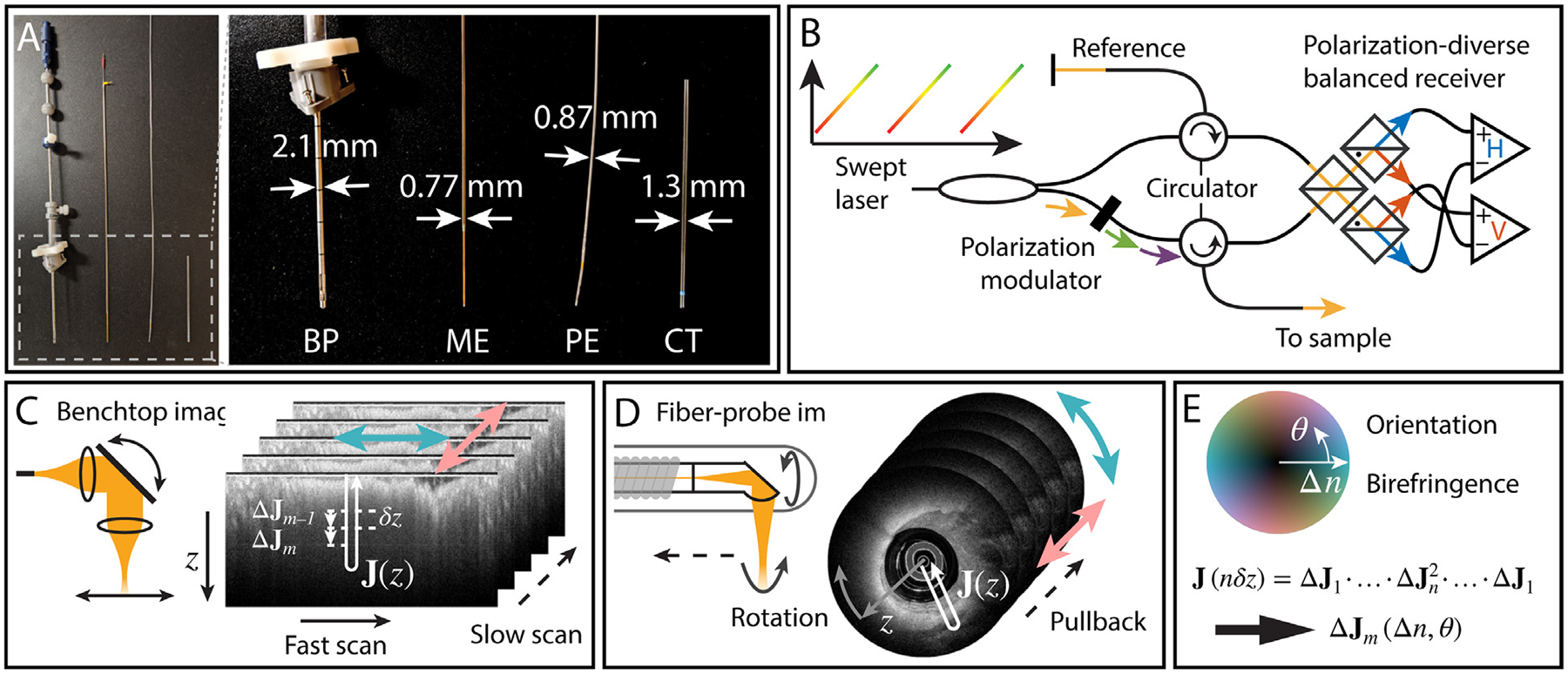
System description and imaging geometry. (A) Photograph of commercial neurosurgical tools, PSOCT endoscope and capillary tube for size comparison. BP: Biopsy probe (Medtronic), ME: microelectrode (Alpha Omega), PE: PSOCT endoscope, CT: capillary tube cannula. (B) Schematic of the PSOCT system used herein. (C) Representative illustration of the benchtop imaging configuration, wherein 3D information is derived from a fast scanning galvanometer mirror and a slower translation stage. The schematic also depicts the round-trip nature of the measured cumulative Jones matrix **J**(*z*), (white arrow) consisting of sequential transmission Δ**J**_*m*_ through individual tissue layers of one pixel thickness *δ*z, and indicates the color-coded optic axis orientations in the image coordinates (colored double-arrows). (D) Representative illustration of the endoscopic imaging configuration, wherein 3D information is derived from a continuous rotation of the side-looking imaging probe combined with a translational “pullback” motion. (E) Birefringence and its optic axis orientation are derived through iterative reconstruction from cumulative round-trip matrices and displayed using a combined color hue and brightness map. The brightness maps the birefringence range of 0–1.3 × 10^–3^.

Contrary to transmission-based modalities, OCT images the subsurface microstructure of tissue by measuring elastically backscattered light, making it well-suited for medical in vivo imaging (Huang et al., 1991). OCT provides depth-resolved reflectivity profiles without axially scanning the focus, enabling relatively compact imaging probes. Such probes have been shown to provide a convenient imaging geometry for endoscopy, with clinical applications in interventional cardiology (Bouma et al., 2017) and gastroenterology (Tsai et al., 2017). The size of current intravascular OCT probes matches closely that of common neurosurgical tools, as illustrated in Fig. 1A, suggesting the suitability of catheter-based OCT for intracranial imaging. Performing PSOCT through intravascular OCT catheters, we have developed intravascular polarimetry to investigate the polarization properties of coronary atherosclerotic lesions in patients (Otsuka et al., 2020). Our advanced signal processing compensates for the polarization effects of transmission through the rotating probe and preceding tissue layers to recover depth-resolved birefringence and optic axis orientation (Villiger et al., 2018). In comparison, previous PSOCT studies of the brain investigated the averaged, i.e., cumulative, retardance of superficial tissue layers of ∼100 μm in thickness, which complicates identification of smaller or interweaving tracts.

Here, we explore the demonstrated polarization contrast of WMT with our advanced reconstruction algorithms and in an imaging geometry that is compatible with intracranial imaging during neurosurgery. We present deep-brain endoscopic PSOCT imaging of WMT in fresh, intact, porcine brain tissue and confirm the reconstructed depth-resolved polarization contrast by comparing WMT orientation using both benchtop and endoscopic PSOCT systems.

## 2. Materials and methods

### 2.1. PSOCT

OCT detects the small fraction of light backscattered by tissue interferometrically through combination with a strong reference signal. Use of a broad spectrum of wavelengths permits coherence gating and achieves high resolution axial imaging of subsurface tissue structure. Throughout this work, we used custom-built, all fiber-based, optical frequency domain imaging (OFDI) systems as described by Yun et al. (Yun et al., 2006).

PSOCT furthermore measures the polarization state of the backscattered light as a function of its round-trip pathlength travelled into the tissue. Our implementation of PSOCT uses polarization-diverse detection in combination with polarization modulation of the light illuminating the sample (Saxer et al., 2000) (Fig. 1B). Consecutive depth-scans (A-lines) are acquired with illumination polarization states that are located orthogonal to each other on the Poincaré sphere. This measurement strategy informs on the overall cumulative retardance experienced by the light on its round-trip propagation through the system components, the rotating probe, and the tissue. It permits the use of standard optical fiber and has been shown to enable reliable and robust polarization measurements in a clinical setting (Villiger et al., 2018).

### 2.2. Benchtop PSOCT system

The system used for benchtop imaging was described in detail by Ren et al. (Ren et al., 2017). Briefly, the light source was a home-built wavelength-swept laser with a center wavelength *λ*_c_ = 1300 nm and a wavelength sweep range of 110 nm. Assuming a Hanning-window for the spectral shape, this resulted in a full width at half maximum of the intensity point spread function, i.e., the squared norm of the reconstructed tomogram of a single reflector, of ∼9 μm in tissue, assuming a refractive index of 1.35. The repetition rate of the laser was 54.4 kHz. Focusing in the lateral directions was achieved using an objective lens (LSM03, Thorlabs), with a spot size specified as 25 *μ*m (Fig. 1C).

### 2.3. Endoscopic PSOCT system

All endoscopic imaging was performed with a custom-built PSOCT console, previously described in (Villiger et al., 2018). The laser characteristics of this system were similar to those of the benchtop system, including an axial width of the point spread function of ∼9 μm, with the exception of an increased repetition rate (103.6 kHz). The imaging console interfaced with a commercial intravascular probe (FastView, Terumo), supporting rotational imaging at up to 100 revolutions per second and featuring a lateral focus spot size of ∼35 μm. In this work a pullback speed of 1 mm/s was used at 50 revolutions per second.

The probe comprises an optical fiber core embedded into a drive shaft to transfer torque, which is encapsulated by an outer protective sheath (Fig. 1D). The outer diameter (OD) of the probe was 870 *μ*m (2.6 Fr) and the angle-polished ball lens at the optical-fiber-tip was designed to image at a focal distance of about 1 mm from the edge of the outer sheath. To mimic a stereotactic neurosurgical procedure wherein rigid metal cannulas are used to descend surgical tools, a rigid, clear capillary tube (Fisherbrand) with an inner diameter (ID) of 1.1 mm and an OD of 1.3 mm was employed to guide the flexible probe. The probe tip, within the capillary can be seen in Fig. 3A, indicated by the white arrow.

### 2.4. Imaging procedure

For the benchtop and correlative endoscopic imaging, intact hemispheres were sliced into coronal sections of about 5 mm in thickness. The tissue slabs were kept on ice for imaging. In the case of intact deep-brain imaging, entire hemispheres were placed in agar molds to maintain the form of the fresh tissue. The agar molds were placed in an ice bath. Fresh porcine brains (LAMPIRE Biological Laboratories, Inc.) were imaged within 32 h of harvest. A total of four porcine brains were used during this study. Shown in the manuscript are representative examples from two separate porcine brains, one for benchtop and correlative endoscopic imaging and one for intact brain imaging.

Importantly, the deployed intravascular probe is flexible, designed for navigation through coronary arteries. In contrast, stereotactic surgery traditionally employs rigid, straight tools to reach deep brain structures. To mimic a rigid probe, we inserted the available flexible imaging probe into the rigid capillary tube (Fig. 1A). For endoscopic deep-brain imaging, the capillary cannula was descended into the brain first, followed by introduction of the probe. Imaging was performed during combined rotation and pullback of the probe. In the presented work, pullbacks took ∼ 30 s to acquire. The pullback speed was chosen to be 1 mm/s to assure optimal image quality. However, clinical uses of the same OCT probe in intravascular procedures routinely involve pullback speeds as fast as 40 mm/s at twice the rotation speed.

Data was acquired with an in-house C++ application software which allowed for real-time structural contrast pre-viewing as well as data streaming to a RAID hard drive array. Data was subsequently post-processed in MATLAB for polarimetric reconstruction.

### 2.5. Depth-resolved birefringence and optic axis reconstruction

Together with conventional tomograms of log-scaled scattering intensity, we reconstructed images of depth-resolved optic axis orientation and nominal birefringence, as described in detail in (Villiger et al., 2018). In short, PSOCT measures the cumulative effect of the round-trip transmission through system components, the rotating catheter, and the sample, which we assume to be accurately described by retardation. The intrinsic round-trip geometry of the measurements constrains this retarder to have linearly polarized eigenstates, allowing to compensate for system components that break this symmetry. Using spectral binning (Villiger et al., 2013), we also compensated for the presence of wavelength-dependent polarization effects in the static system components. We then used the reflection signal from the ball lens at the tip of the catheter to correct for the dynamic transmission through the rotating probe and isolate the sample round-trip transmission. The pathlength-resolved cumulative retardance corresponds to the sequential product of Jones matrices describing the transmission through the linear retarder of individual tissue layers of one pixel thickness, followed by reverse transmission. The local, i.e., depth-resolved birefringence and optic axis orientation of the individual layers are reconstructed iteratively by correcting for the effect of preceding tissue layers (Fig. 1E) and referenced to known optic axis orientations, such as that of the endoscope sheath in the case of endoscopic imaging or discernable WMT in the case of benchtop imaging. The recovered depth-resolved optic axis orientation indicates the direction of the linear polarization experiencing locally the fastest propagation through the tissue. It corresponds to the azimuthal orientation of WMT within the local coordinate system defined by the probing beam originating from the zenith and the scan directions for benchtop imaging (Fig. 1C), and a plane tangential to the cannula surface, using the longitudinal and circumferential directions of the cannula as reference for endoscopic imaging (Fig. 1D).

The scalar amount of birefringence indicates the effective birefringence at each depth. Because areas without birefringence result in random optic axis orientations, we combine the optic axis orientation and scalar birefringence for display, mapping the orientation in color hue and birefringence in brightness. Areas where the measured polarization states are random, either due to insufficient signal to noise ratio or multiple scattering, were automatically identified using the degree of polarization (DOP) and masked out.

## 3. Results

### 3.1. Benchtop PSOCT of fresh porcine brain tissue

**Fig. 2.**
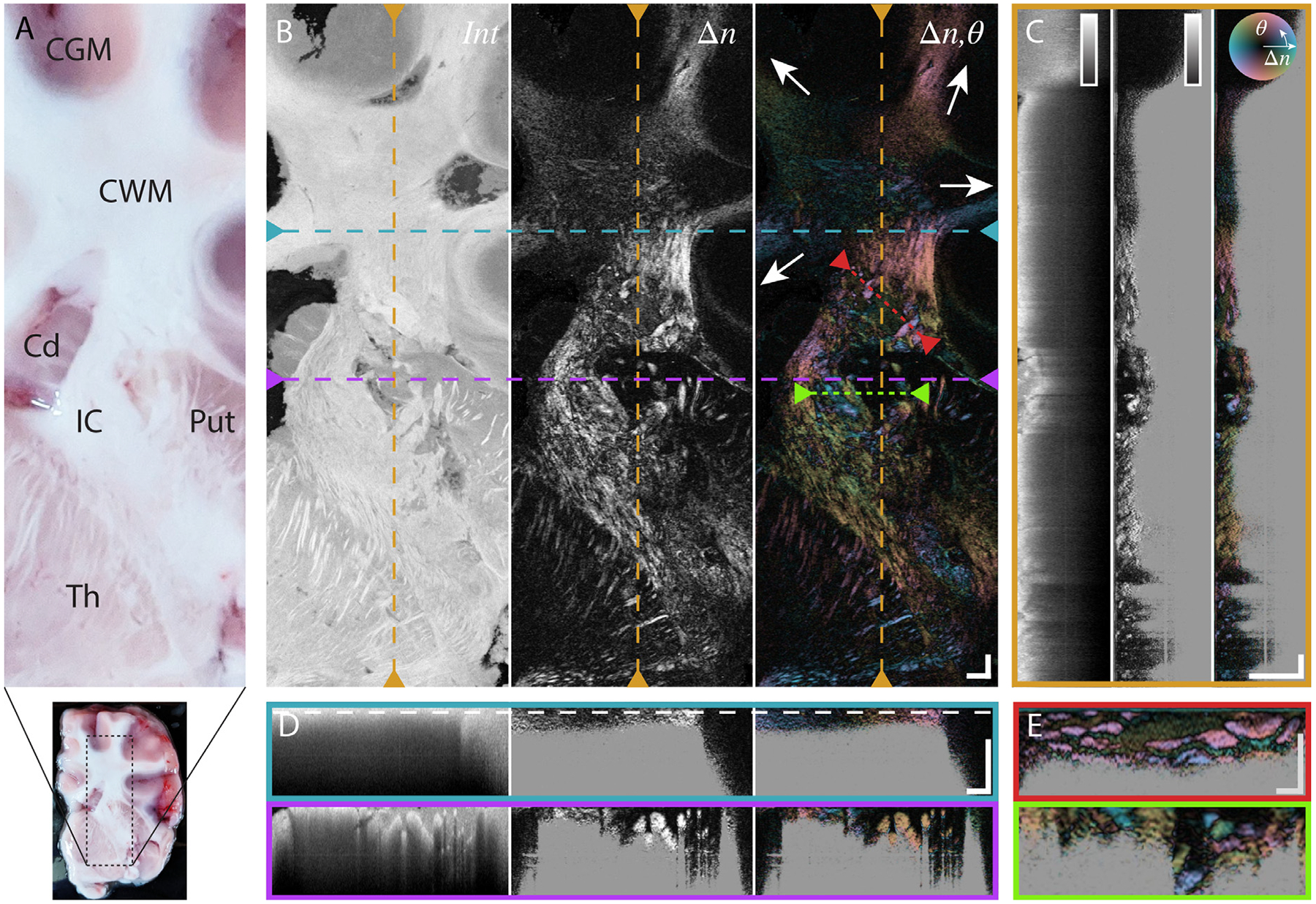
Large FOV benchtop imaging of thick, fresh porcine brain tissue using PSOCT. (A) Reference picture of imaged brain slice with magnified inset, corresponding to PSOCT-imaged area, with labelled anatomical areas. CGM: Cortical Gray Matter. CWM: Cortical White Matter. Cd: Caudate Nucleus. IC: Internal Capsule. Put: Putamen. Th: Thalamus. (B) En-face PSOCT images. Order from left to right: logarithm of scattering intensity, scalar birefringence, optic axis orientation with birefringence brightness overlay. The white arrows highlight the different strands of the WMT structure. Colored dashed lines represent the position of the corresponding cross-sectional slices in the other panels. (C) Cross-sectional slice along the slow scan direction. (D) Cross-sectional slices along the fast scan direction. The white dashed line indicates the depth position of the en-face view in B. (E) Cross-sectional slices highlighting depth-resolved features. Scale bars are 1 mm in general, and 500μm for panel E. Color maps for various contrasts are located as inserts in C and show the range of 0–40 dB for the intensity signal and 0–1.3 × 10^–3^ for birefringence. Images present mean projections computed along the respective out-of-plane directions over 30 μm for en-face and 70 μm for cross-sectional views, centered on the indicated locations.

We first investigated the ability to achieve polarization contrast within fresh brain tissue by performing benchtop PSOCT imaging on large, thick slabs of porcine brain. Shown in Fig. 2 are the results of such imaging, providing cross-sectional and *en-face* images of contrast from conventional OCT scattering intensity, scalar birefringence, as well as optic axis orientation. All of the presented contrasts are reconstructed from a single-acquisition volumetric data set.

As expected, WMT scatter more strongly in comparison to gray matter brain regions. Furthermore, the same structures show much higher birefringence. The optic axis follows the expected direction of the WMT, highlighted by the white arrows in Fig. 2B, easily perceived given the large FOV of the benchtop imaging system. Importantly, while previous work has performed polarimetric analysis of the cumulative retardance signal in serially sectioned brain tissue, here we present the first depth-resolved optic-axis imaging of WMT, best appreciated in cross-sectional views (Fig. 2C and D). The center of the WMT star-shaped region of the cortex features lower birefringence than expected, likely due to interweaving WMT below the resolution of the system, or an out-of-plane orientation of the WMTs, which would reduce the observed birefringence. The optic axis provides information on the azimuthal orientation of the WMT within the imaging plane, even where the traditional OCT contrast shows a uniform scattering signal without any directional information, such as within the cortical white matter. Throughout the white matter portions of the brain, WMT can be better appreciated in the birefringence and optic axis signals than in the intensity signal. Only small WMTs entirely surrounded by gray matter (e.g., in the thalamus) allow clear visualization of strands of nerve fibers by the intensity contrast. Lastly, Fig. 2A presents a camera-phone image of the same brain slab, for visual reference.

### 3.2. Comparing endoscopic PSOCT to benchtop imaging

**Fig. 3.**
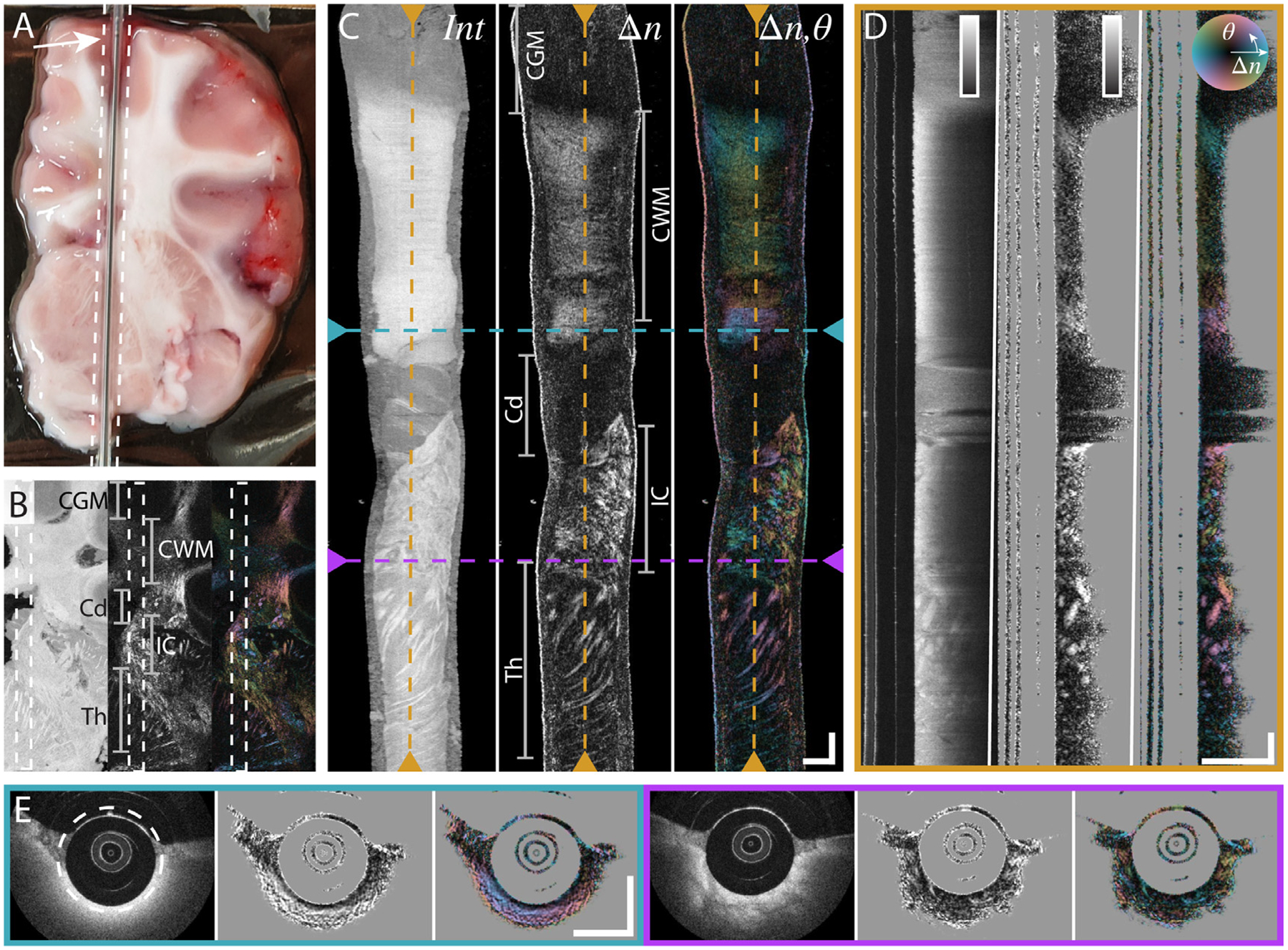
Comparing PSOCT contrast between benchtop microscope and small form-factor rotational endoscope. (A) Camera image of brain slice with probe inside capillary in place, before initiating imaging pullback. White arrow highlights probe-tip within capillary. (B) Preceding benchtop en-face image from Fig. 2 for reference. White dashed lines correspond to endoscopic imaging area. Gray bars indicate anatomical areas: CGM: Cortical Gray Matter. CWM: Cortical White Matter. Cd: Caudate Nucleus. IC: Internal Capsule. Th: Thalamus. (C) Unfolded en-face view of PSOCT contrasts imaged with endoscopic probe. Order from left to right: logarithm of scattering intensity, scalar birefringence, optic axis orientation with birefringence brightness overlay. Colored dashed lines represent the position of the corresponding cross-sectional slices in D and E. Gray bars highlight same anatomical areas as in B. (D) Cross-sectional slice along the pullback direction. (E) Cross-sectional slices along the rotational direction. White dashed circle indicates position of unfolded en-face image in C. All scale bars are 1 mm. Color maps for various contrasts are located as inserts in D and show the range of 0–40 dB for the intensity signal and 0–1.3 × 10^–3^ for birefringence. Images present mean projections computed along the respective out-of-plane directions over 30 μm for en-face, 2.5° for cross-sectional pullbacks, and 140 μm for cross-sectional rotational views, respectively, centered on the indicated locations.

To compare the endoscopic PSOCT signal to benchtop imaging, the same brain slab was subsequently imaged from within a transparent capillary cannula positioned on top of the slab, within the former experiment’s imaging area. This method of open-face imaging with the endoscope allowed for visual confirmation of the acquisition location with both the camera image as well as the benchtop OCT experiment. Combined benchtop and endoscopic imaging was performed in several tissue slabs, and a representative example is shown in Figs. 2 and 3.

As with the benchtop imaging experiment, in Fig. 3 we see that the WMT imaged with the endoscope have both higher scattering intensity and birefringence. Furthermore, for the first time we show that optic axis contrast from WMT in fresh brain is achievable with a probe-based system. The rich information provided by the optic axis contrast in regions of uniform scattering intensity are further highlighted in Fig. 3D and 3E. While direct pixel-to-pixel correlation of benchtop and endoscopic PSOCT remained difficult due to tissue deformation between experiments, by eye, we can easily co-register the endoscopic information with the benchtop OCT data, in particular the area of cortical WMT at the beginning of the pullback and the WMT in the internal capsule and extending into the thalamus.

### 3.3. Deep brain PSOCT in fresh porcine brain

**Fig. 4.**
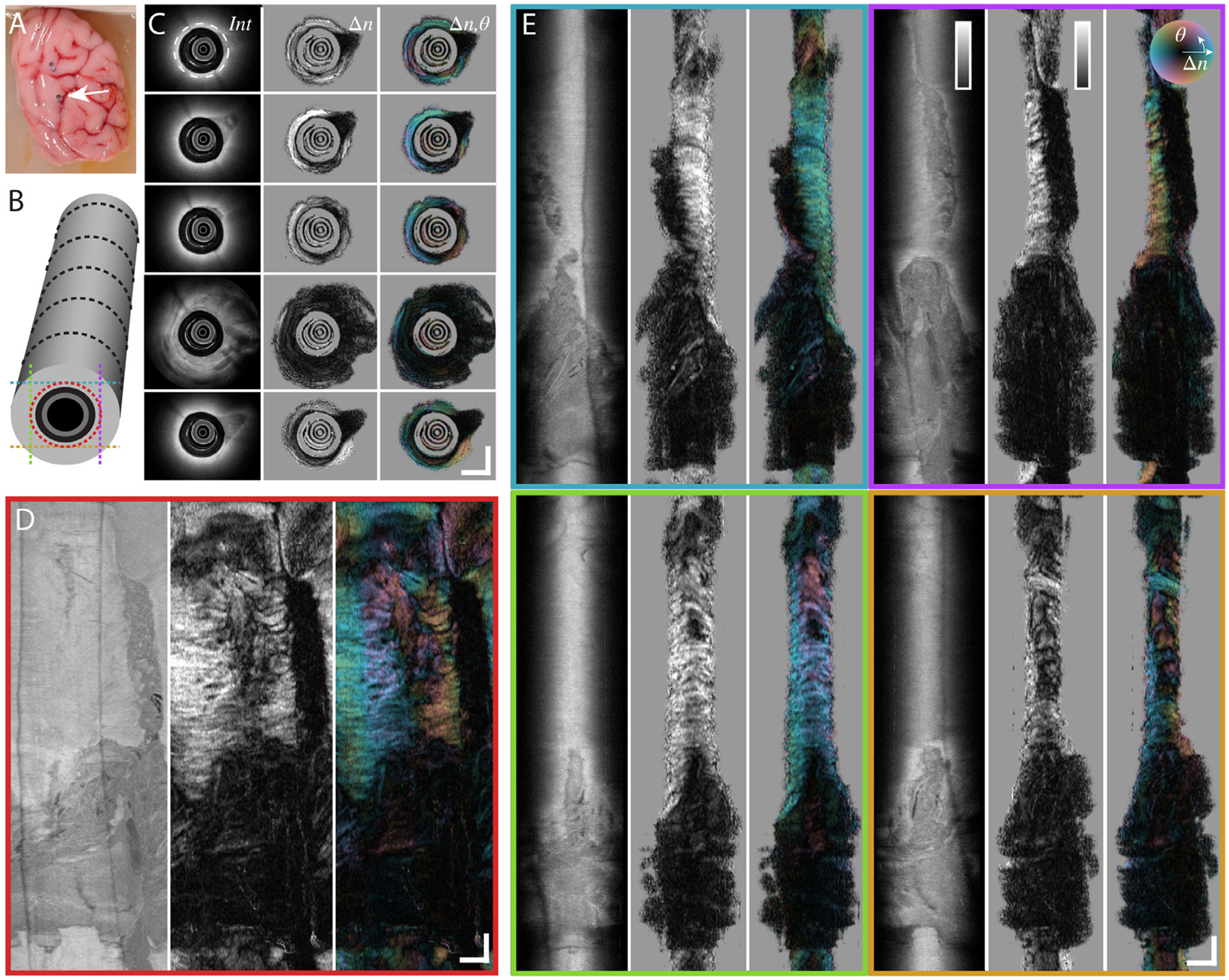
Deep brain PSOCT in intact, fresh, porcine brain. (A) Reference image of capillary tube cannula inserted into the right hemisphere of the fresh brain tissue, used to guide imaging probe descent. (B) Schematic legend for corresponding 2D slice locations within 3D imaging volume. Black lines represent different depths of cross-sectional rotational slices in C, and red circle and colored lines indicate locations of unwrapped *en-face* view in D and of tangential cross-sections in E, respectively. (C) Rotational cross-sections at various positions along pullback. Top image is deepest in the brain. Order from left to right: logarithm of scattering intensity, scalar birefringence, optic axis orientation with birefringence brightness overlay. (D) Unwrapped *en-face* view at constant distance from the capillary surface. Position of en-face image is indicated by dashed white circle in first intensity cross-section of C. (E) Tangential cross-sections within imaging volume. All scale bars are 1 mm. Color maps for various contrasts are located as inserts in E and show the range of 0–40 dB for the intensity signal and 0–1.3 × 10^–3^ for birefringence. Images present mean projections computed along the respective out-of-plane directions over 60 μm for cross-sectional rotational views, and 30 μm for en-face and tangential cross-sectional views, respectively, centered on the indicated locations.

As a final proof of concept, we performed deep brain imaging with the miniature endoscope in two intact, fresh, porcine brains. The capillary cannula was used to define a straight imaging trajectory, mimicking stereotactic cannulas. Fig. 4 shows the scattering and polarization contrast from a representative pullback located in the central region of the right hemisphere. Unlike benchtop imaging, where large FOV *en-face* images of scattering intensity can offer intuitive interpretation of WMT orientation, the local information provided by endoscopic imaging is much harder to comprehend. Indeed, the interpretation is complicated by the helical scan pattern which provides local information confined to a cylindrical region surrounding the cannula. As seen in Fig. 4, scattering intensity alone is able to differentiate gray matter from white matter but provides little information on WMT alignment or orientation. While the scalar birefringence offers slightly improved insight in comparison to scattering intensity, suggesting organization of white matter into individual tracts, optic axis orientation provides the clearest demarcation of individual WMT as well as their orientation. This ability offers improved identification of distinct tissue regions that generate a homogenous scattering signal and may add important geometric information for localizing the position of a deep brain instrument.

## 4. Discussion

This work explores the potential of endoscopic PSOCT for deep brain imaging and neurosurgical guidance. We first showed benchtop PSOCT imaging of fresh brain tissue, demonstrating that recent advancements with depth-resolved PS processing enable reconstruction of axially resolved birefringence and its optic axis orientation over millimeter depths. Previous uses of PSOCT in the brain were limited to superficial imaging of cumulative retardance in fixed brain tissue. Subsequently, probe-based imaging of the same brain section confirmed consistency with benchtop data, revealing close agreement between the birefringence and optic axis of WMT imaged in the overlapping fields of view. Finally, we showed deep brain imaging over centimeter long probe pull-backs using our endoscopic system. The rich birefringence contrast alludes to the wealth of navigational information PSOCT could provide deep within the brain, for instance in the localization of a deep brain instrument in reference to WMT landmarks.

OCT uses optical radiation at biologically safe levels, as shown in various other clinical applications cited earlier. The system is all-fibered, portable, and robust to movement, having already been used in the clinic for human cardiovascular imaging. The intravascular probe presented has an outer diameter of 870 *μ*m, making it smaller than routine tools sent deep into the brain, such as chronic electrodes for deep brain stimulation (DBS) and biopsy cannulas for tissue collection. Furthermore, similar probes with outer diameters of less than 400 *μ*m have already been developed in our lab and can be adapted for this application (Tan et al., 2012). As a further practical advantage, the endoscope length can be made to exceed 2 m, allowing for the instrument console to be kept outside of the sterilized surgical zone. Importantly, while imaging was performed at 1 mm/s pullback rates, faster rotation and pullbacks are routine in intravascular imaging and are therefore envisioned for the future of this application. Moreover, the use of capillary tubes was exemplary, and directly designing rigid probes may be more practical.

Other optical methods have been proposed for neurosurgical guidance based on spectroscopy (Giller et al., 2009; Johansson et al., 2009; DePaoli et al., 2018; DePaoli et al., 2019), laser Doppler flow (Wårdell et al., 2016) and traditional OCT (Jafri et al., 2009). While endoscopic OCT has recently been shown for imaging neural tissue in mice (Yuan et al., 2020) and detecting blood vessels in humans (Ramakonar et al., 2018), traditional OCT contrast is limited in WMT. The PSOCT system described here adds to this growing body of work and separates itself by offering the capability to image WMT structure and orientation. In comparison to other imaging and spectroscopy techniques, OCT provides depth resolved imaging with no focus-altering optics at imaging depths up to millimeters in gray matter and hundreds of microns in white matter. This feature allows for smaller imaging probes with larger imaging areas.

A focus application for this technology may be the guidance of neuromodulation procedures where implantation accuracy is critical. Indeed, WMT are often indicative landmarks for deep brain nuclei, or even stimulation targets themselves (Prent et al., 2019; Vanegas-Arroyave et al., 2016). The most common neuromodulation procedure, subthalamic nucleus (STN)-targeted DBS for Parkinson’s Disease (PD), often uses microelectrode recordings (MER) to help localize the target region after pre-operative planning. The use of MER is not unanimous, however, due to conflicting reports on the advantages (Giller and Jenkins, 2015; Lee et al., 2018; Lozano et al., 2019). Furthermore, other emerging targets for DBS (both for PD and other conditions) cannot make use of MER due to non-characteristic neuronal activity in the surrounding regions. Moreover, optimal stimulation location for chronic electrodes remains debated, even for STN-DBS which has been studied extensively (Hamel et al., 2017). Finally, factors such as brain shift (Khan et al., 2008) and poor MRI resolution (Starr et al., 2002; Starr, 2002) can generally limit the accuracy of DBS procedures. The proposed PSOCT system could serve both as research tool to aid in clarifying optimal functional targets as well as a conduit for surgical guidance to improve implantation accuracy by providing intraoperative feedback on the electrode’s true location in reference to WMT landmarks. dMRI and tractography are emerging as useful tools for pre-surgical planning. Specific examples of its utility have been shown through improved tumor resection margins (Essayed et al., 2017; Costabile et al., 2019) and locating optimal targets for DBS (Prent et al., 2019; Calabrese, 2016; Hunsche et al., 2013; Horn et al., 2017). Importantly, dMRI may be ideally complemented by intraoperative endoscopic PSOCT. Pre-operative imaging and planning making use of WMT architecture to improve surgical targeting could benefit from high-resolution optical imaging for in situ registration. Similar pre- and intra-operative imaging fusion across modalities has been shown between MRI and ultrasound (Prada et al., 2014). In the case of surgical resection procedures dMRI has shown to be advantageous for localizing eloquent areas; however, direct electrical stimulation (DES) remains the gold standard for the intraoperative mapping of these structures. Local PSOCT endoscopy in combination with global dMRI imaging may provide a promising tandem to spare such structures without the pitfalls of DES, which include the requirement for awake surgery and the possibility of seizure (Essayed et al., 2017).

Ultimately, all optical modalities provide relatively local information in comparison to MRI and dMRI, which can provide whole brain architecture. Even with the improved optical penetration depth that has been shown with OCT systems using lasers operating at 1.7 *μ*m (Chong et al., 2015), it is apparent that extensive computational efforts will be required to correlate the local optical information provided by PSOCT with classical surgical planning modalities.

Furthermore, we present here a method to perform azimuthal, in-plane “apparent” optic axis imaging. The retardance experienced by the probing beam indeed depends on the alignment between its propagation direction and the three-dimensional optic axis orientation of the tissue, defining an additional ‘out-of-plane’ angle, separate to the azimuthal optic axis orientation, that currently remains undefined. Full 3D orientation mapping can be achieved by imaging the sample under various angles, as used by PLI (Axer et al., 2011) and some benchtop PSOCT implementations (Ugryumova et al., 2006). To properly correlate endoscopic PSOCT with dMRI, this out-of-plane component would be helpful. Full 3D optic axis mapping may be achieved algorithmically by leveraging the intrinsic catheter rotation, which defines a continuously changing illumination angle, or via new probe designs that include multiple, angularly diverse imaging channels.

## 5. Conclusion

The birefringence of myelinated axons can serve as an intrinsic contrast mechanism for the visualization of WMT, as validated extensively with PSOCT and PLI in *ex vivo* benchtop studies. Here we showed how advanced PS processing has enabled the same contrast mechanism for imaging the location and orientation of deep WMT through narrow-gage probes, compatible with clinical intracranial imaging. Our endoscopic OCT system is fiber-based, portable, and robust to movement, allowing swift clinical translation. In neuromodulation procedures, such as DBS, the relative position of stereotactic trajectories to surrounding WMT are of critical interest and this system may provide valuable new insights for optimal electrode placement and perhaps aid in guiding implantations in the future.

## Data Availability

An open-source Python package for reconstruction of conventional OCT tomograms and depth-resolved tissue birefringence is available on http://github.com/CBORT-NCBIB/oct-cbort. The authors encourage additional inquiries and will be happy to provide additional Matlab scripts for reconstruction of depth-resolved optic axis orientation and compensation of catheter-transmission. The raw OCT measurements are also available upon request.
